# Recent advances in aortic valve replacement

**DOI:** 10.12688/f1000research.17995.1

**Published:** 2019-07-22

**Authors:** Cristiano Spadaccio, Khalid Alkhamees, Nawwar Al-Attar

**Affiliations:** 1 Department of Cardiac Surgery, Golden Jubilee National Hospital, Agamemnon Street, Glasgow, G81 4DY, UK; 2 Department of Cardiac Surgery, Prince Sultan Cardiac Center Al Hassa, Prince Fawaz bin Abdulaziz St., Hofuf city, 31982, Saudi Arabia

**Keywords:** aortic valve, surgery, replacement

## Abstract

Aortic valve replacement has stood the test of time but is no longer an operation that is exclusively approached through a median sternotomy using only sutured prostheses. Currently, surgical aortic valve replacement can be performed through a number of minimally invasive approaches employing conventional mechanical or bioprostheses as well as sutureless valves. In either case, the direct surgical access allows inspection of the valve, complete excision of the diseased leaflets, and debridement of the annulus in a controlled and thorough manner under visual control. It can be employed to treat aortic valve pathologies of all natures and aetiologies. When compared with transcatheter valves in patients with a high or intermediate preoperative predictive risk, conventional surgery has not been shown to be inferior to transcatheter valve implants. As our understanding of sutureless valves and their applicability to minimally invasive surgery advances, the invasiveness and trauma of surgery can be reduced and outcomes can improve. This warrants further comparative trials comparing sutureless and transcatheter valves.

## Introduction

The realm of aortic valve replacement (AVR) is quickly changing. The increasing use of transcatheter techniques and the advancement of sutureless valve are contributing to a change in both the indications and the operative strategies for AVR, particularly for patients with aortic stenosis (AS). AVR is indicated in symptomatic patients with severe stenosis (mean pressure gradient of at least 40 mm Hg or maximum velocity of at least 4 m/s) or in asymptomatic patients with impaired left ventricular ejection fraction or low surgical risk ^1^. With the publication of the Cavalier trial ^2^ of the Perceval sutureless aortic valve and the Placement of Aortic Transcatheter Valves (PARTNER) 2 randomised controlled trial ^3, 4^, the spectrum of treatment of aortic valve disease has definitely increased. In this review, we will focus mainly on the most important recent advances in the surgical field, including the incoming surgical valve technologies, such as the sutureless devices, and surgical accesses to the aortic valve, such as minimally invasive surgery via hemisternotomy or right minithoracotomy.

## Standard aortic valve replacement: the “classic” surgical technique and its results

Standard aortic valve replacement (SAVR) is the classic commonly used approach for aortic valve surgery and is performed through a median sternotomy with cardiopulmonary bypass with excellent outcomes. Following transverse or hockey-stick aortotomy, the pathological valve leaflets are excised and the annulus is debrided. A series of interrupted sutures (with or without pledgets) or continuous sutures are placed under direct vision to anchor a biological valve, whether stented or stentless, or mechanical valve. Whilst mechanical prostheses suffer extremely rarely from structural valve deterioration and have excellent durability, the rates of freedom from structural valve failure in stented bioprostheses are 70 to 90% at 10 years and 50 to 80% at 15 years ^5^. However, incoming manufacturing technologies such as the one used for the new Resilia Inspiris valve (Edwards Lifesciences, Irvine, CA, USA) are meant to significantly increase bioprosthesis durability and the results of the Prospective, non-randomized, Multicenter Clinical Evaluation of Edwards Pericardial Bioprostheses With a New Tissue Treatment Platform (COMMENCE) trial are eagerly awaited ^6^.

Given the outstanding short- and long-term outcomes, SAVR is deemed to be the gold standard operation for aortic valve disease and represents the benchmark against which new therapies are compared ^7^. Also, SAVR remains the only option in several hostile conditions such as endocarditis, anomalies of coronary origin, bicuspidy or redo surgery after homograft implantation in the congenital population.

Improvements in the perioperative management of critically ill elderly patients with multiple comorbidities have widened the range of patients eligible for surgery. Interestingly, in a 2015 review of 141,905 patients undergoing isolated first-time SAVR between 2002 and 2010, the majority of patients were in a surgical low-risk group (80% low risk, 13.9% intermediate risk, and 6.2% high risk) ^8^, suggesting that this category still constitutes the greatest part of patients undergoing treatment. When the operative mortality was stratified by using the Society of Thoracic Surgeons (STS) predictive risk classification, actual in-hospital mortality was significantly lower in all patients (2.5% versus 2.95%) both in the overall population and within each risk category. Of note, there was a notable increase in the percentage of high-risk (STS of more than 8%) and intermediate-risk (STS of 4 to 8%) patients undergoing surgery from the earlier to the latter years (from 5.7 to 6.6% and 12.8 to 14.9%, respectively).

However, certain subgroups of patients with significant comorbidities (lung disease, renal insufficiency and so on) and deemed at elevated risk might not be considered suitable candidates for SAVR and are currently the main point of discussion at heart team meetings. In addition to significant comorbidities and excessive preoperative risk, SAVR may be declined in otherwise-fit patients presenting anatomical features that determine particular intra-operative challenges (that is, porcelain aorta, small aortic annulus or previous chest radiotherapy). These shortfalls in SAVR have stimulated the development of alternative interventions in the form of sutureless aortic valve replacement (SuAVR), minimally invasive aortic valve replacement (MIAVR) and, more recently, transcatheter aortic valve implantation/transcatheter aortic valve replacement (TAVI/TAVR).

## Sutureless aortic valve replacement: technique and results

Whilst conventional surgical AVR performed by median sternotomy still represents the standard of care in the treatment of aortic disease, less invasive approaches are progressively gaining consensus by providing effective results with a reduced interventional burden on the patient. Sutureless (rapid deployment) valves are bioprostheses that can be surgically implanted without the need of anchoring sutures (or not more than four annular anchoring sutures ^9^) as in the traditional fashion while still allowing complete excision of the diseased native valve and cleaning of aortic annulus of calcified debris or infected material.

Two main types of sutureless aortic prostheses categorised by implantation mechanism are available on the market ^10^: the self-expandable Perceval S (Sorin, Saluggia, Italy) and the balloon-expandable Intuity (Edwards Lifesciences) sutureless valve. Sutureless valves are amenable to be implanted by median sternotomy or minimally invasive accesses such as ministernotomy (MIS) and right anterior thoracotomy (RAT) ^11^. These approaches have been shown to reduce bleeding and blood transfusions, atrial fibrillation, wound infection, ventilation times, and time to return to normal activities ^12, 13^.

Indications for sutureless valve implantation equate to those for surgical AVR ^14^. Sutureless valves can be applied routinely but are particularly pertinent in patients with multiple comorbidities or those in need of multiple procedures by its ability to reduce cross-clamp time. Hanedan *et al*. showed better haemodynamic outcomes and shorter ischaemic times in elderly, high-risk patients who underwent multiple cardiac surgical procedures when implanted with sutureless valves rather than conventional AVR ^15^. The significant reduction in time of implantation makes the sutureless valve a valuable adjunct in the case of double-valve procedures or need for concomitant atrial fibrillation ablation, as shown recently by Baran *et al*. ^16^.

An International Expert Consensus Panel recommends sutureless valves as first choice of valve prosthesis for patients who require concomitant procedures or who have a small aortic annulus. Further indications are for the patient who requires a redo operation or who has a delicate aortic wall condition ^17^.

The main drawback of sutureless valves concerns paravalvular leaks and the need for pacemaker implantation. The Surgical Treatment of Aortic Stenosis With a Next Generation Surgical Aortic Valve (TRITON) study with the Edwards Intuity prosthesis demonstrated a paravalvular leak rate at 1 year of 2.3% (1.4% and 0.9% for early and late occurrences, respectively) ^18^.

The reported incidence of pacemaker implantation ranges from 5.6 to 9.1% in the literature ^19, 20^, worse than standard AVR (3.0%). Other significant complications include neurological events (transient ischemic attack or disabling stroke), myocardial infarction, kidney failure, and surgical site infections ^21^.

Another recent multi-centre retrospective study compared the outcomes of Perceval S and Intuity valves in a propensity-matched analysis ^22^. Little difference was found in the rate of pacemaker implantation: 6% in the Perceval S group and 6.8% in the Intuity group, respectively; similar early clinical and haemodynamic outcomes were reported. Perceval S was associated with shorter aortic cross-clamp and cardiopulmonary bypass times (52 ± 14 minutes in the Perceval S group versus 62 ± 24 minutes in the Intuity group), but transaortic peak and mean gradients were lower in the Intuity group (mean gradients of 11.8 ± 4.7 in the Perceval S group versus 10.5 ± 3.9 in the Intuity group) ^22^.

## Minimally invasive aortic valve replacement

### Minimally invasive surgery: the techniques

MIAVR is defined as an AVR procedure that, as opposed to conventional full sternotomy, is performed through a small chest wall incision ^23^. Two techniques are available and amenable to be paired with the use of sutureless valves: MIS and RAT ^11^.

The first approach entails a “J” sternotomy performed at the level of the third or fourth intercostal space which provides direct access to the aorta and, in the majority of the cases, to the right atrium, permitting the cardiopulmonary bypass to be established centrally via the ascending aorta and right atrium.

The second approach foresees a skin incision of 5 to 7 cm placed at the level of the second intercostal space to access the chest cavity. Care is taken to avoid rib spreading, although some reports describe the need for rib resection. Direct aortic cannulation can be performed with a flexible cannula while venous drainage is achieved via the femoral vein by using a multi-stage cannula positioned into the right atrium with the Seldinger technique and under transoesophageal echocardiographic guidance. As described by Glauber *et al*., accurate planning of this procedure with the use of multi-slice computed tomography is required with the aim to evaluate the anatomical relationship among intercostal spaces, the ascending aorta and aortic valve ^24^. Also, transverse aortotomy is normally performed 2 cm higher than conventional aortotomy; the rest of the procedure is carried out in accordance with the instructions of the manufacturer. Briefly, suture guidance is used to accompany the prosthesis and then the valve is deployed or balloon-expanded according to the model used ^24, 25^.

### Minimally invasive surgery: the clinical results

A number of meta-analyses have revealed several advantages in using a minimally invasive approach, including reduction of bleeding and transfusion requirement and postoperative complications, such as atrial fibrillation, wound infection and ventilation time, leading to an overall shortening of the length of stay in hospital ^12^. The benefits in the postoperative management of these patients further translate to a quicker return to daily activities with fewer costs related to rehabilitation resources. Minimally invasive AVR can be performed via MIS or RAT; the latter produces more evident benefits ^24, 26^. Sutureless valve technology seems the best fit in this context where the risk profile of the intervention meets the technical challenge of restricted access with more complex angles of chest entrance and field visualisation.

Minimally invasive approaches to the aortic valve have also been used in the redo setting. Besides the expected technical challenges of redo surgery though a minimal access, the major concern relies in the possibility to deliver an adequate myocardial protection strategy. Also, in the case of redo after coronary surgery, the limited surgical field might render the isolation and control internal thoracic artery (ITA) grafts prior to clamping extremely challenging ^27^. In a meta-analysis ^27^ of small retrospective studies, no significant differences in in-hospital mortality and stroke (ranging from 0 to 9.5% and 2.6 to 8%, respectively) were detected when comparing minimally invasive approaches with the aortic valve and conventional sternotomy in re-operative settings. Similarly, no significant difference was found in the length of hospital stay or rates of pacemaker implantation, renal failure, re-operation for bleeding, and hospital stay between the two groups. Vola *et al*. showed the feasibility of using minimally invasive SuAVR in three patients with degenerated small 19 mm aortic bioprostheses with no mortality and an average implantation time of 10.3 minutes ^28^. However, the current body of evidence relies mainly on outcomes from experienced centres with scarce possibility of diffuse worldwide reproducibility of these results.

### Minimally invasive surgery versus standard surgery

Several studies comparing the outcomes of RAT with median sternotomy demonstrated lower incidence of postoperative atrial fibrillation, blood transfusion, and shorter ventilation time and hospital length of stay in the RAT group ^24^. Also, it has been demonstrated that RAT might be a valid adjunct in the treatment of octogenarians or elderly patients and a more expeditious and effective alternative to full sternotomy AVR. In a report by Gilmanov *et al*., RAT was associated with lower postoperative stroke incidence, earlier extubation and shorter hospital stay ^29^.

In a large retrospective study with RAT, sutureless valves outperformed standard suturable valves through the same approach. Although 1-year mortality, incidence of postoperative strokes and pacemaker implantation rate were similar in the two cohorts, cardiopulmonary bypass and cross-clamping times were significantly shorter in the sutureless group and postoperative duration of mechanical ventilation was also reduced. Interestingly, a larger prosthesis could be implanted in the sutureless valve group ^30^. In another retrospective study, by Beckmann *et al*., implantation of sutureless valves in small aortic annulus patients achieved effective orifice areas comparable to patients receiving root enlargement surgery and conventional AVR ^31^. However, there was no difference in 30-day mortality and survival rates and no statistically significant increase in the incidence of patient–prosthesis mismatch ^31^. These findings are pertinent, especially when the risk–benefit balance of performing AVR in high-risk or geriatric patients with small annuli is considered.

Borger *et al*. ^32^ published a multi-centre randomised trial comparing MIAVR using the Edwards Intuity valve versus full sternotomy SAVR in 46 and 48 patients, respectively. Sutureless valve replacement was associated with significantly lower cross-clamp durations (41.3 versus 54 minutes), mean transvalvular gradients (8.5 versus 10.3 mm Hg) and prevalence of patient–prosthesis mismatch (0% versus 15.0%) at 3 months. This study was underpowered to investigate differences in mortality or morbidity; however, no clear differences in early clinical outcomes, including quality-of-life measures, were found. Pacemaker implantation rates were higher in the sutureless cohort but this was not statistically significant (4.3% versus 0%). Previous non-randomised studies confirmed shorter procedural times, which did not translate to better outcomes, and showed comparable in-hospital mortality and perioperative stroke rates ^30, 33^. Some investigators reported lower rates of blood transfusions, shorter intensive care unit (ICU) and intubation times, and lower incidences of postoperative atrial fibrillation and respiratory insufficiency with SuAVR and this translated to significant overall cost reductions that were attributed mainly to reduced overall hospital stay and diagnostics ^33^. Despite this positive evidence in favour of MIS, a comprehensive meta-analysis in 2017 failed to show a significant advantage in clinical outcomes with the exception of a reduction in postoperative stay and blood consumption and concluded with the need for randomised evidence to elucidate this point ^34^.

The results of two randomised trials have recently been published. The Mini-Stern trial was a randomised clinical trial comparing full sternotomy with MIS for AVR. The trial failed to show shorter hospital stay and faster recovery or improved survival and was not cost-effective. It was concluded that the MIS approach is not superior to full sternotomy for performing AVR ^35^.

More recently, the MAVRIC (manubrium-limited ministernotomy versus conventional sternotomy for aortic valve replacement; ISRCTN29567910)—a single-centre, single-blind randomised study—compared AVR via manubrium-limited ministernotomy using a 5 to 7 cm midline incision (intervention) and conventional median sternotomy and had postoperative red cell transfusion as the primary outcome. MIS was associated with higher cardiopulmonary bypass time and reduced drain losses but this difference did not translate in a significant reduction in blood transfusion. Additionally, conventional SAVR was found to be more cost-effective (MIS had a 5.8% probability of being cost-effective at a willingness to pay of £20,000 per quality-adjusted life year) ^36^. Evidently, the benefit of MIS over full sternotomy is still debated and the adoption of one or the other approach is driven mainly by the experience of each centre.

Generally, one of the main obstacles to the wide adoption of minimally invasive AVR is the association with increased operative times, technical difficulty and steep learning curves. Interestingly, a meta-analysis revealed a weighted mean difference of 7.9 additional minutes of cross-clamp time with minimally invasive AVR ^12^; however, more randomised evidence is needed to be the final word on this debate.

### Minimally invasive surgery: comparison among the different techniques

Similarly, RAT outperformed MIS in terms of postoperative complication and length of stay in hospital ^11^. Interestingly, a 2015 report from Hassan *et al*. examining the cost-effectiveness and logistic balance in MIS and RAT-AVR from eight large studies in the US—while showing no significant inter-group differences in 30-day mortality, conversion to sternotomy, neurologic events, arrhythmia, wound infection, or postoperative bleeding among the two groups—demonstrated slightly better outcomes in terms of blood transfusion and hospital stay ^37^. A subsequent cost-effectiveness analysis demonstrated that, given a volume of 50 cases per year, the added operative costs per case were US$ 4,254 for RAT-AVR and US$ 290 for MIS-AVR. The added costs per case, assuming 200 cases per year, were US$ 4,209 and US$ 290, respectively. A RAT-AVR programme performing 50 cases per year adds US$ 1,063,665 of operative costs over five years compared with US$ 72,500 for a MIS-AVR programme. Unlike the results of previously reported studies, these results suggest that the clinical benefits of MIS-AVR are comparable to or better than those of RAT-AVR and cost less, prompting careful consideration in healthcare delivery organisations when developing minimally invasive surgical valve replacement programmes ^37^.

## Transcatheter aortic valve replacements: results and comparison with surgery

Since its first application in humans by Cribier *et al*. ^38^, TAVR has experienced a progressive expansion in indications and a rapid technological development. TAVR was initially targeted at patients who have severe AS and are unfit for conventional surgery ^39– 42^, and in our previous report ^43^, we highlighted the procedural techniques and important trials in this field. The most recent follow-up of these initial trials suggested a progressive shift in indications, including intermediate-risk categories.

Two large multi-centre trials—Surgical Replacement and Transcatheter Aortic Valve Implantation (SURTAVI) ^44^ and PARTNER 2A ^3^—have indeed demonstrated non-inferiority of TAVI versus SAVR for treatment of severe AS in patients at intermediate surgical risk. Compared with SAVR, the SURTAVI also showed that percutaneous technology produced better haemodynamics and significantly lower rates of all stroke at 30 days, acute kidney injury and atrial fibrillation.

Finally, in low-risk patients, the latest results of the PARTNER study series demonstrated lower rates of death or stroke and new-onset atrial fibrillation in TAVR than surgery at 30-day follow-up, and the composite of death, stroke or rehospitalisation at 1 year significantly favoured TAVR over surgery. Also, no significant differences in major vascular complications, new permanent pacemaker insertions, or moderate or severe paravalvular regurgitation were found among the two groups ^45^.

These results should be weighted against the still-unknown long-term durability of TAVR. Patient preference and individual patient factors, such as age and small left ventricular outflow tract, and other factors not normally included in the currently used surgical scoring systems play a significant role. Additionally, compared with TAVR, SAVR continues to have absolute lower rates of residual paravalvular leakage, major vascular complications and new permanent pacemakers ^22^ (reported as ranging from 13.2 to 17.1%) ^46, 47^. Lastly, this randomised evidence arises from the comparison of the most modern TAVR technology with old-fashioned conventional AVR through median sternotomy. The recent literature has been intrigued by the idea of comparing TAVR with the use of a similarly advanced technology on the surgical side (that is, sutureless valves) within the context of a minimally invasive approach. Sutureless valves with a minimally invasive approach would still have the advantage to remove the diseased valve, thus achieving an adequate calcium debridement of the annulus with potential to implant more haemodynamically performing valves and reduce complications such as paravalvular leaks, one of the main predictors of poor survival ^48^, constituting a valid surgical alternative to TAVI ^49^. Three meta-analyses focusing on comparisons among sutureless valves and TAVI and conventional prosthesis have been performed ^25, 50, 51^. A significant reduction in mortality and complications in the perioperative period was found in the sutureless valve group. The low- and intermediate-risk population benefitted from a reduction of at least 30% in 30-day mortality and in the risk for paravalvular leak ^51^, and Takagi *et al*. confirmed better survival in sutureless valve–AVR over TAVI after combining the results of direct-comparison and adjusted indirect-comparison meta-analyses ^50^. However, when a comparison with conventional AVR was made, outcomes were burdened by similar mortality but a reduced rate of permanent pacemaker implantation ^51^. These results confirm the findings of a previous meta-analysis including 5,000 high-risk patients in which sutureless valve–AVR produced a reduction in early mortality and postoperative paravalvular leak ^52^ and echo the data from Biancari *et al*. in intermediate-risk patients ^53^. One of the most recent reports eventually demonstrated better perioperative mortality, 1- and 2-year survival, and paravalvular leak occurrence and similar ICU length of stay, pacemaker implantation need, and kidney failure in the sutureless group ^25^. The SuAVR group was troubled by increased transfusion requirements, whereas TAVR was hampered by major vascular complications ^25^. Interestingly, when the results of these studies comparing MIS sternotomy sutureless AVR with TAVR were specifically examined, better rates of paravalvular leakage and improvement in survival at 2 years could be demonstrated in the surgical group, suggesting that minimally invasive sutureless valve–AVR could be considered as the first-line treatment for high-risk patients in the “grey zone” between TAVR and conventional surgery ^54^. Similarly, RAT sutureless valve–AVR showed a significant reduction in paravalvular leaks and a trend towards improved immediate- and mid-term outcomes and survival when compared with TAVR ^55^.

In terms of cost-effectiveness, the literature initially produced discouraging data regarding the reimbursement of TAVR showing a lack of economic advantage in its clinical use ^56^. However, at a later stage, the results of the longer-term follow-up on TAVR allowed a more permissive view on the applicability of TAVR, even on intermediate low-risk candidates ^57– 59^. Again, these results derive from a comparison of TAVR and conventional AVR. The results of a head-to-head randomised comparison between the most modern transcatheter technology and access and the relative counterpart in the surgical field are eagerly awaited.

Given the latest evidence regarding the progressive expansion of indications for TAVI, the choice between transcatheter and surgical AVR is continuing to incite debate among heart team members. In this context, SUV-AVR provides an interesting alternative, especially for high-risk patients with borderline indications or contraindications for TAVI.

## Conclusions

The face of AVR is rapidly changing in both the interventional and surgical fields. In the surgical realm, the introduction of sutureless valve technologies, obviating the need for anchoring sutures, has been shown to reduce operative time and duration of the cardiopulmonary bypass, being amenable to be applied to combined cardiac surgery procedures (that is, AVR and coronary bypass grafting, double-valve procedures, and so on). The use of these devices also simplifies minimally invasive approaches and is a valid adjunct in patients with small aortic annulus or fragile aortic wall or requiring redo operations. Increased use of these valves in current surgical practice should be considered by the heart team and encouraged ^60^. However, there is a paucity of long-term durability data in contrast to conventional stented bioprostheses and mechanical valves, which still represent the gold standard for the surgical treatment of aortic valve disease. New incoming technologies combined with minimally invasive approaches will surely achieve more importance in surgical practice, but SAVR remains a cornerstone in “non-conventional” conditions such as redo surgery after homograft or autograft implantation, congenital structural abnormalities, bicuspidy and endocarditis.

## Abbreviations

AS, aortic stenosis; AVR, aortic valve replacement; ICU, intensive care unit; MIAVR, minimally invasive aortic valve replacement; MIS, ministernotomy; PARTNER, Placement of Aortic Transcatheter Valves; RAT, right anterior thoracotomy; SAVR, standard aortic valve replacement; STS, Society of Thoracic Surgeons; SuAVR, sutureless aortic valve replacement; SURTAVI, Surgical Replacement and Transcatheter Aortic Valve Implantation; TAVI, transcatheter aortic valve implantation; TAVR, transcatheter aortic valve replacement
